# Mediterranean Journal of Rheumatology June 2018 Issue Highlights

**DOI:** 10.31138/mjr.29.2.65

**Published:** 2018-06-29

**Authors:** Lazaros I. Sakkas

**Affiliations:** Department of Rheumatology and Clinical Immunology, Faculty of Medicine, School of Health Sciences, University of Thessaly, Larissa, Greece

In this issue of MJR there are interesting case reports, research protocols and reviews.

Panagopoulos et al.^1^ reviewed the role of microRNAs (miRNAs) in the pathogenesis of osteoarthritis (OA) and their potential role as therapeutic targets in this disease. miRNAs are small, single-stranded non-coding RNAs that regulate gene expression at the post-transcriptional level.

Chikanza et al.^2^ use a case of a woman with Adamantiades-Behcet disease (ABD), who subsequently developed monoclonal gammopathy of unknown significance (MGUS) to speculate on the mechanisms of development of MGUS and ABD. ABD shares features of auto-immune disease and autoinflammatory disease and the authors elaborate that an early event in B cells, such as IgH translocation, may make them sensitive to growth factors, such as interleukin(IL)-6 which is raised in ABD. Also, the CD56 marker, increased in ABD T cells, is also increased in MGUS plasma cells.

Patients with antiphopsholipid syndrome (APS) may develop angina and myocardial infarction. In a research protocol, Tektonidou et al.^7^ will utilize stress perfusion cardiac magnetic resonance in asymptomatic patients with APS to detect myocardial ischemia.

Tsalapaki et al.^8^ in a 5-year prospective protocol will study disease course, comorbidities, treatment efficacy and outcome in giant cell arteritis in Greece.

O’Brien et al.^9^ in a research protocol will examine longitudinal relationships between sedentary behavior (defined as waking behavior characterized by ≤ 1.5 metabolic equivalents, METS) while in a sitting, reclining or lying position, or light intensity physical activity(1.6-<3.0 METS) with health outcomes in rheumatoid arthritis.

Calcified chest lymph nodes in a patient with systemic sclerosis (SSc) is a rare finding in SSc. Yet, as Angelopoulou et al.^6^ pointed out, this finding as well SSc may well be a consequence of silica exposure.

Migkos et al.^5^ reported on two patients with Sjogren’s syndrome (SjS) who developed polymyositis and inclusion body myositis and identified another 24 cases of SjS with inflammatory myopathies in the literature.

Venetsanopoulou et al.^3^ reported on a patient with ankylosing spondylitis who developed systemic sclerosis (SSc) and scleroderma renal crisis. The co-existence of ankylosing spondylitis and SSc is rare.

Patients on immunosuppressants are susceptible to various infections. In this issue, Kostopoulos et al^4^ described a patient with RA treated with steroids, methotrexate and adalimumab developed Orf disease, also known as ecthyma contagious; a rare self-limited disease caused by a DNA virus of the parapoxvirus group and transmitted to humans from goats and sheep.
